# Regional gray matter volume mediates the relationship between neuroticism and depressed emotion

**DOI:** 10.3389/fpsyg.2022.993694

**Published:** 2022-10-06

**Authors:** Junyi Yang, Xiaoyang Huangfu, Dandan Tong, Anming He

**Affiliations:** ^1^School of Education Science, Xinyang Normal University, Xinyang, China; ^2^School of Psychology, Northwest Normal University, Lanzhou, China

**Keywords:** neuroticism, depressed emotion, longitudinal tracking, voxel-based morphometry, dmPFC

## Abstract

The underlying psychological mechanism of the effect of neuroticism on depressed emotion has been widely studied. However, the neural mechanism of this relationship remains unclear. Therefore, the present study aimed to apply voxel-based morphometry (VBM) to explore the neural mechanism of the relationship between depressed emotion and neuroticism in healthy and young participants through longitudinal tracking research. The behavioral results showed that neuroticism was positively related to depressed emotion at T1 and T2 (6 months later). The VBM analysis revealed that neuroticism positively associated with the gray matter volume (GMV) in the dorsal medial prefrontal cortex (dmPFC). Mediation analysis was conducted to investigate the neural basis of the association between depressed emotion and neuroticism. The mediation result revealed that GMV of the dmPFC partially mediates the relationship between neuroticism and depressed emotion at T1 but not T2. Together, these findings suggest that the gray matter volume of dmPFC could may affect the relationship between depressed emotion and neuroticism.

## Introduction

Depression is typically characterized by loss of interest, depressed mood or various behavioral, physiological, cognitive, and affective symptoms (Sabah et al., 2019). Depressed emotion is typically characterized by low mood, but this mood is short-lived and can be resolved without treatment, although a continuously low mood easily induces depression (Penninx et al., 2003). In healthy people, depressed emotion is one of the most common emotional problems and has an important impact on individual physical and mental health. Previous research has revealed that neuroticism is an important factor affecting depression, and neuroticism is associated not only with clinical patients’ depressive symptoms but also with nonclinical patients’ depressed emotions (Farmer et al., 2002; Yang et al., 2016b; Mu et al., 2020; Sami and Naveeda, 2021). A study of 900 healthy individual subjects revealed a significant and positive association between neuroticism and depressed emotion (Jylhä and Isometsä, 2006). A longitudinal follow-up study found that neuroticism can reliably predict the depressed emotions among individuals (Fanous et al., 2007; Yao et al., 2009; He et al., 2018).

Why do individuals with high neuroticism have higher level of depressed emotion? At present, research on the relationship between neuroticism and depressed emotion is more focused on exploring psychological mechanisms among healthy samples. For example, researchers have found that shame may be an important psychological variable to explain neuroticism and depressed emotion (Paulus et al., 2016). Additionally, emotion dysregulation can also be used to explain the potential psychological mechanism of neuroticism and depressed emotion (Yoon et al., 2013). Moreover, other researchers suggested that neuroticism may not directly affect depressed emotion levels, but instead may indirectly affect depressed emotion by affecting the individual’s cognitive processing, such as automatic thinking (Du et al., 2015). However, the relationship between behaviors is complex, and the relationship between neuroticism and depressed emotion may involve not only psychological factors but also physiological mechanisms. However, at present, the neural mechanism between neuroticism and depressed emotion is not clear. In a previous study of healthy subjects, neuroticism scores were positively correlated with resting hippocampal activation intensity in female subjects, and hippocampal activation intensity mediated the relationship between neuroticism scores and depressive symptom scores (Sutin et al., 2010). Additionally, exploring whether the influence of neuroticism on depressed emotion has a stable neural basis is of great significance for the improvement of negative emotions in the future and the improvement of individual mental health levels. Thus, the neural mechanism of the relationship between depressed emotion and neuroticism among healthy subjects was investigated in present study.

Neuroticism is a personality that is correlated with negative experiences or effects that significantly affect or impact human life, behavior, and communication (Yang et al., 2021). With the development of magnetic resonance technology and the advantages of individual differences research, the individual differences in the brain mechanism of neuroticism can be studied. For example, in a study of 116 healthy adults, neuroticism scores negatively correlated with gray matter volume (GMV) in the medial prefrontal cortex (mPFC; DeYoung et al., 2010). Another study revealed a negative correlation between neuroticism and GMV in the hippocampus and the dorsal medial prefrontal cortex (dmPFC) among 87 healthy elderly subjects (Kapogiannis et al., 2013). Additionally, a previous study of 35 healthy subjects revealed that neuroticism positive correlated with GMV in the anterior cingulate cortex (ACC) in women, while the opposite effect was found in men (Blankstein et al., 2009). Furthermore, a meta-analysis of the structural changes in the brain with neuroticism revealed that individuals with high levels of neuroticism had a larger GMV in the left amygdala (Mincic, 2015); although a negative correlation has also been reported (Omura et al., 2005).

The current investigation aimed to explore the relationship between depressed emotion and neuroticism and to elucidate the underlying neural mechanism of this effect. Specifically, the individual differences in young and healthy participants were examined. For this purpose, voxel-based morphometry (VBM) was used to examine the association between the volume of brain regions and neuroticism. Based on previous findings about neuroticism, we hypothesized that neuroticism might affect the GMV of prefrontal cortex linked to cognitive–emotional functions (Ochsner and Gross, 2005), as well as the amygdala, which play important roles in emotional behavior (Whittle et al., 2014). A previous study showed that depression was associated with increased GMV in medial prefrontal regions (Li et al., 2017) and with decreased GMV in amygdala (Zavorotnyy et al., 2018). Thus, we hypothesized that the GMV of some regions related to neuroticism may mediate the relationship between neuroticism and depressed emotion.

## Materials and methods

### Participants

All the participants of the present study were part of our ongoing project to examine the associations between emotion, brain imaging, and mental health. The participants of this study were either graduate or undergraduate students from the Southwest University (China). Participants with a psychiatric history of neurological disorders were not included in the present study. All participants provided informed consent prior to this study. Study approval was obtained from the Review Board of Brain Imaging Center Institutional of Southwest University (China).

A total of 359 participants (159 male) completed the personality trait scale and underwent MRI. The self-rating depression scale (SDS) was administered approximately 1 week after the MRI (T1). Only 342 of the 359 participants completed the personality trait scale, the SDS and MRI. In the present study, 172 of the 342 participants completed the SDS again at 6 months (T2).

### The neuroticism personality trait

The Neuroticism-Extraversion-Openness Personality Inventory (NEO-PI-R) was used to measure the neuroticism. The NEO-PI-R includes 240 items. The NEO-PI-R is based on the five-factor personality model and includes six subscales (Costa and McCrae, 1992; Yang et al., 2016a). Previous studies suggested this tool have high validity and reliability (Costa and McCrae, 1992; Yang et al., 2021). A total of 48 items were used to assess neuroticism; a five-point Likert scale was used for each item, with response options ranging from strongly disagree to strongly agree. We used the total scores for neuroticism; thus, the neuroticism scores ranged from 48 to 240 in this study.

### The self-rating depressed emotion scale

Depressed emotion was measured by the SDS. This questionnaire contained 20 items, responses for each item were provided on a four-point Likert-scale ranging from “rarely” to “frequently.” Individuals has higher scores on the SDS indicated has higher levels of depressed emotion (Zung, 1965). The SDS is widely used to measure depressed emotion in healthy individuals and have high validity and reliability (Zung, 1971; Zhang et al., 2012; Wang et al., 2015). We used the total scores of the SDS as the score of depressed emotion; thus, total scores ranged from 20 to 80.

### Data acquisition through MRI

The 3.0-T Siemens Trio-MRI-scanner was used for MRI tests. The T1-weighted (high-resolution) anatomical images (echo time = 2.52 ms, flip angle = 9-degree, repetition time = 1,900 ms, inversion time = 900 ms, resolution matrix = 256 × 256, thickness = 1.0 mm, slices = 176, and voxel size = 1 × 1 × 1 mm^3^) were obtained by a magnetization-prepared rapid gradient echo (MPRAGE) sequence.

### Structural data preprocessing

The SPM8 was used to process the MRI data. For better registration, we used an SPM8-based script that was automatically coregistered to the anterior commissure-posterior commissure (AC-PC) of all T1-weighted structural images. Then, we used the VBM8 toolbox to perform a spatially adaptive nonlocal means (SANLM) denoising filter. Next, the coregistered images of each participant were segmented into gray matter (GM), white matter (WM), and cerebrospinal fluid by using the unified segmentation procedure. A diffeomorphic nonlinear registration algorithm (DARTEL; diffeomorphic anatomical registration through exponentiated lie algebra) was used to spatially normalize the data to a study-specific T1-weighted template of GM images for each participant. The DARTEL registration involves the following steps. First, the specific template based on the average tissue probability maps from all the participants was computed; Second, segmented maps of each participant were warped to a specific template. To improve the alignment and to achieve a more accurate inter-subject registration, the procedure was repetitively conducted until the best study-specific template was generated. Then, registered images were transformed to the Montreal Neurological Institute (MNI) space, and to preserve the volume of the GM, further modulation was performed. Finally, the modulated GM images were smoothed using a 10-mm full width at half maximum (FWHM) Gaussian kernel.

### Statistical analyses

The brain imaging data were analyzed by SPM8. Regression analysis was performed to determine the association between neuroticism and GMV. In this regression model, total brain GMV, age, and sex were entered as covariates. Neuroticism scores were entered as variables of interest. To limit the search volume to white and gray matter, explicit masking in SPM8 was applied, using the population-specific masking toolbox. This method replaces absolute or relative threshold masking and limits the false negative risks that can be caused by overly restrictive masking. It may not be included in the analysis as potentially interesting voxels (Ridgway et al., 2009).

To increase the power to detect individual differences in neuroticism and brain structure, the analyses focused on some key brain structures associated with neuroticism. Thus, in present study, we used a small volume correction (SVC) to correct for multiple comparisons (Worsley et al., 1996; Becker et al., 2015). According to previous findings, the dmPFC, mPFC, ACC, amygdala, and hippocampal gyrus were chosen as regions of interest (ROIs). Structural regions of these ROIs were defined using the WFU Pickatlas Toolbox (Maldjian et al., 2003). Individual differences within the ROIs were computed using a threshold of *p* < 0.05 (family-wise error-corrected, FWE; minimum cluster size > 100 voxels; Yang et al., 2017).

### Mediation analysis

To determine the role of regional GMV in the relationship between depressed emotion and neuroticism, mediation analysis was performed in the present study. In the mediation model, the mediating variable “M” affects the causal effect of an independent variable “X” on a dependent variable “Y.” The indirect macro was used in SPSS (Preacher and Hayes, 2008) to conduct the mediation analyses. In the present study, “M” was the GMV of regions, “Y” was the SDS score, and “X” was neuroticism. The corrected cluster result for each ROI was significantly correlated with neuroticism. The volume of the ROI signals from each of the subjects was determined by the SPM8 toolbox. A previous study revealed that age and sex affect GMV (Gennatas et al., 2017); thus, in this model, the total GMV, sex, and age were used as covariates. The bias corrected confidence intervals (CI = 95%) were registered. A significant indirect effect is represented by a non-zero value, which indicates that the independent variable has an effect on the dependent variable through the mediator variable (Preacher and Hayes, 2008).

## Results

### Behavioral results

Table 1 shows the demographic characteristics of the subjects and the association between SDS scores (T1 and T2) and neuroticism. As shown in Table 1, when controlling for age and gender, the partial correlation analysis showed that neuroticism was positively related to SDS scores at T1 (*n* = 342) and T2 (*n* = 172). The results revealed that the subjects with higher levels of neuroticism had a higher level of depressed emotion, and neuroticism can be used to predict individual SDS scores 6 months later.

**Table 1:** The characteristics of demographic information about the large young sample.

Items	Mean	*SD*	Relation to neuroticism (*r*)
Age (*n* = 359)	20.00	1.32	
Neuroticism (*n* = 359)	138.66	19.17	
Age (*n* = 342)	19.97	1.27	
Neuroticism (*n* = 342)	139.37	18.44	
Depression emotion (T1, *n* = 342)	43.42	8.66	0.607^***^
Depression emotion (T2, n = 172)	44.28	9.93	0.418^***^

*** *p* < 0.001.

### Correlation between GMV and neuroticism

**Figure 1:**
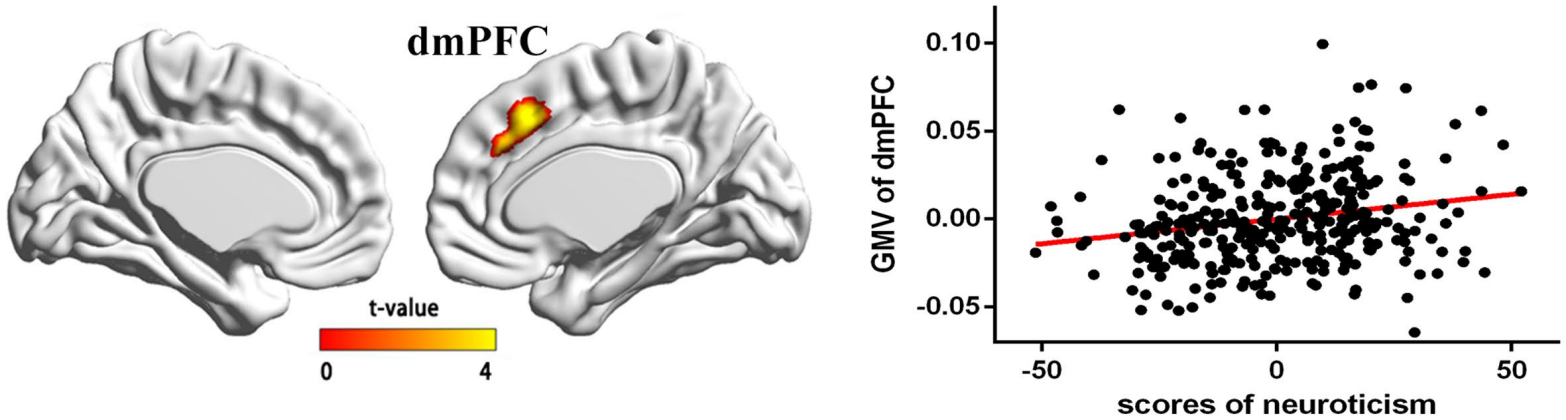
The regions of positive association between gray matter volume (GMV) and neuroticism (the results are *p* < 0.05, corrected) and the partial correlations (control age, sex, and whole brain gray volume) in a scatterplot between neuroticism and mean GMV within a significant cluster mainly included areas in the dmPFC (*x* = 5, *y* = 22, and *z* = 44).

In the regression analysis (*n* = 359), age, global GMV, and gender were entered as covariates, and neuroticism was entered as the variable of interest. The results revealed a significantly positive correlation between neuroticism and GMV in the dmPFC (*t* = 4.25; *x* = 5, *y* = 22, *z* = 44; cluster size = 567 voxels; *p* (SVC) < 0.05; Figure 1).

To test whether GMV of dmPFC was associated with the neuroticism, we saved dmPFC as ROI and subsequently extracted the GMV of dmPFC from each participant using the SPM8 toolbox. Next, we tested the relationships between the GMV of dmPFC and the neuroticism using SPSS 16.0. After controlling for sex, total gray matter volume, and age, the results showed that the neuroticism scores were positively correlated with the GMV of dmPFC (see the scatter plot in Figure 1).

### Mediation results

**Figure 2:**
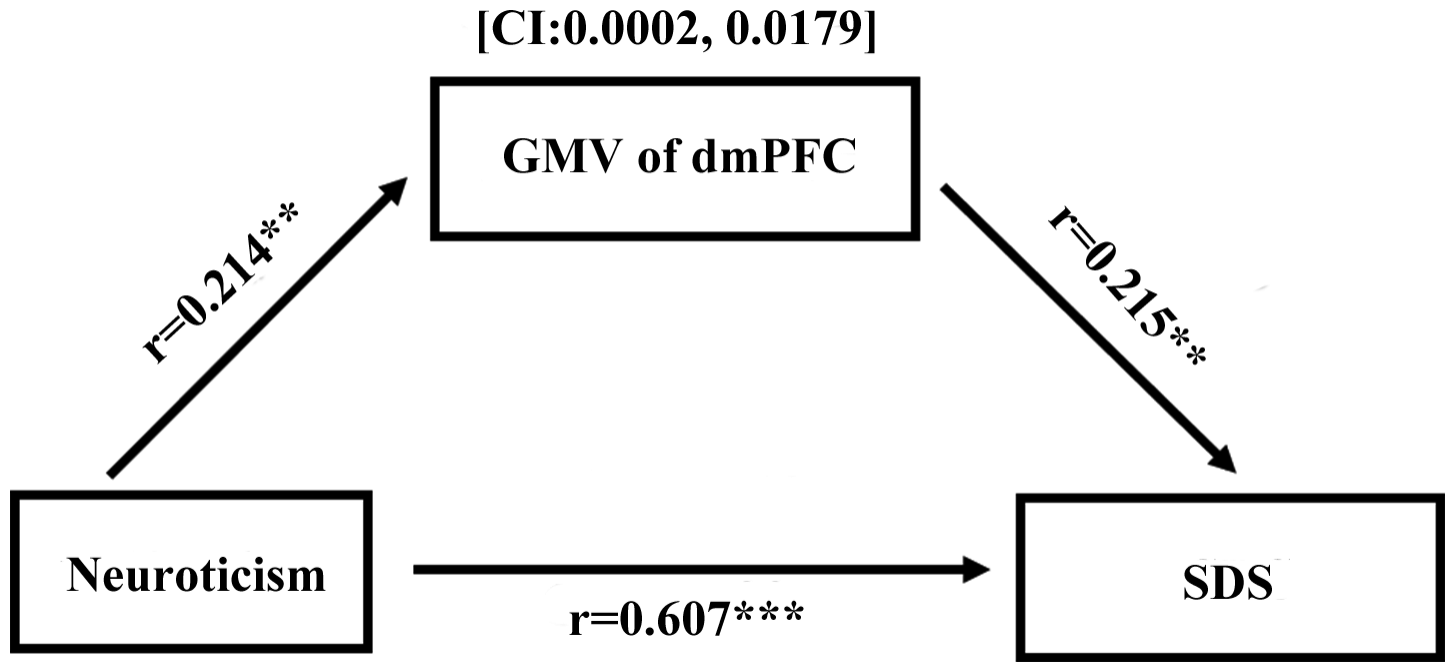
Mediation analysis revealed that the GMV of dmPFC could partly mediated the relationship between depressed emotion and neuroticism (^**^p < 0.01, ^***^p < 0.001).

After controlling for age, sex, and global GMV (*n* = 342), neuroticism was positively associated with the volume of the dmPFC (*r* = 0.214, *p* < 0.01). Additionally, neuroticism was positively correlated with SDS (for T1, *r* = 0.607, *p* < 0.001; for T2 *r* = 0.418, *p* < 0.001). Furthermore, the volume of the dmPFC was positively associated with the SDS (for T1, *r* = 0.215, *p* < 0.01; for T2, *r* = 0.189, *p* < 0.05). To test the significance of the indirect effect between depressed emotion and GMV of the dmPFC, bootstrap resampling was used. The results showed that the GMV of the dmPFC partially mediates the relation between SDS and neuroticism (*c* = 0.272, *c*’ = 0.263, CI: 0.0002, 0.0179; see Figure 2) at T1. Conversely, the analysis revealed a nonsignificant and indirect effect of dmPFC volume on the relationship between neuroticism and SDS at T2 (CI: −0.007, 0.035).

The results showed that only the dmPFC was a significant mediator; it could be that the correction was set during the first step of looking for brain regions associated with neuroticism. Other regions that did not meet the correction criteria were ignored. We further analyzed our data (*p* < 0.005, cluster size > 100, uncorrected) and found that neuroticism was negatively associated with the GMV of the ACC (*x* = 0, *y* = −2, *z* = 30, *t* = −3.8). However, the mediation analysis showed that ACC does not significantly mediate the relationship between neuroticism and SDS at T1 and T2.

## Discussion

In present study, longitudinal study was used to examine the influence of neuroticism on depressed emotion and investigate the underlying neural mechanism of this relationship in young, healthy individuals through a mediation analysis. The analysis of behavioral results showed that neuroticism could predict individual depressed emotion levels 6 months later. According to the VBM results, neuroticism was positively associated with GMV in the dmPFC. Additionally, the results of the mediation analysis showed that the volume of the dmPFC mediated the relationship between neuroticism and depressed emotion at T1.

The results revealed that neuroticism was positively associated with depressed emotion at T1 and T2. Some previous studies have revealed that neuroticism can predict the depression severity of symptoms, onset, progression, and prognostic effects (Brown, 2007). Similarly, longitudinal studies have also found that neuroticism is still predictive of depressive symptoms 2 years later (Van Loey et al., 2014). Additionally, some studies reported that depression scores are positively and significantly associated with neuroticism among healthy subjects (Yoon et al., 2013; Yildirim et al., 2022). Another longitudinal study found that neuroticism is consistently associated with depressed emotion in a sample of healthy college students (Yao et al., 2009). Some investigations have revealed that depressed individuals with impaired emotion regulation have greater immersive cognitive coping styles (Joormann and Gotlib, 2010). Individuals with high levels of neuroticism have higher levels of cognitive immersion coping style and more negative self-related thinking, which are clinical characteristics of depression (Barnhofer and Chittka, 2010). Therefore, in conclusion, the neuroticism found in this study is stably positively associated with individual depressed emotion, which may be because individuals with higher levels of neuroticism have more inappropriate coping styles and impaired emotion regulation, leading to a higher level of depressed emotion.

The present results also found that neuroticism was positively related to GMV in the dmPFC. In recent years, researchers have proven that the dmPFC associated with emotional processing and emotional regulation (Lungu et al., 2015; Scharnowski et al., 2020; Yang et al., 2020). For example, researchers have found increased activity in the dmPFC of individuals during emotional processing, especially negative emotional processing (Etkin et al., 2011). Other studies have found that in the process of negative emotion regulation, especially in the process of top-down regulation, the main role of the dmPFC is to inhibit the activation intensity of some brain regions of the limbic system, such as hippocampus, hypothalamus, and the amygdala, which are related to feeling emotions, and then to carry out successful emotion regulation (Banks et al., 2007; Yang et al., 2020). The dmPFC is an important component of the default-mode network (DMN)(W. Li et al., 2014) and associated with negative self-reference processing (Herbert et al., 2011; Shiota et al., 2017). Although previous VBM studies did not find neuroticism is positive associated with GMV in the dmPFC, some studies found that during emotion-cognitive task processing the individuals with greater neuroticism scores had abnormal activity in the dmPFC (Yang et al., 2020). Recently, researchers have found evidence refuting the idea that bigger is always better – smaller gray matter volumes have been shown to be associated with better task performance (Kanai and Rees, 2011; Takeuchi et al., 2011). For example, one study found that a smaller ventrolateral prefrontal GMV was associated with greater self-referential ability, while a larger dorsal anterior cingulate GMV was associated with more negative emotional experiences (Takeuchi et al., 2014). Previous study suggested that GMV in the prefrontal cortex, is pruned to some extent during normal development (Kanai and Rees, 2011; Takeuchi et al., 2011; Yang et al., 2020), and the better those regions of the brain are trimmed, the better they function. Taken together, individuals with high levels of neuroticism may be more self-referent, more likely to perceive negative emotions, and have poor emotional regulation.

Contrary to the hypothesis, we did not find significant correlations of neuroticism scores with the GMV of the ACC, amygdala, mPFC, and hippocampus. While some studies have reported associations of neuroticism scores with the GMV of some brain regions, others have not found significant associations of neuroticism scores with the GMV of any brain region. For example, a study with 65 healthy adults found that neuroticism scores did not significantly correlate with the GMV of the amygdala and prefrontal regions (Cremers et al., 2011). In addition, a longitudinal study that followed 274 healthy subjects for 6 years investigated the relationships of neuroticism scores and the GMV of brain regions; the authors found that neuroticism scores did not significantly correlate with the GMV of any brain region (Taki et al., 2013). In addition, we believe other factors explain the discrepancy in the associations of neuroticism scores with the GMV of the mPFC, ACC, amygdala, and hippocampus. First, studies differed in sample sizes, from small (Omura et al., 2005; Blankstein et al., 2009) to relatively large (Taki et al., 2013). Second, studies differed in age ranges, from wide (Cremers et al., 2011; Taki et al., 2013) to narrow (Blankstein et al., 2009; Kapogiannis et al., 2013). The age range of our study was relatively narrow: all subjects were college students or graduate students. Third, studies used different analysis methods, such as ROI analysis (Blankstein et al., 2009; Cremers et al., 2011) or global brain analysis (Taki et al., 2013). Fourth, studies used different corrections, such as SVC (Blankstein et al., 2009) or family wise error (FWE; Taki et al., 2013). We used a small volume correction, but when we relaxed the correction criteria, we found that neuroticism scores were correlated with the GMV of the ACC.

The correlation analysis showed that the depressed emotion was positively related to GMV in the dmPFC. Patients with depression tend to pay more attention to negative stimuli and show more systematic errors in cognitive and emotional processing (Lemogne et al., 2009). Previous investigations have also demonstrated the vital role of the dmPFC in negative self-reference processing, and depressed patients show higher levels of self-preference and self-focus, which is one of the typical features of depression, than healthy controls (Northoff et al., 2006). Other investigations have demonstrated that the dmPFC is an essential node of the DMN, which plays an important role in emotional processing and emotion regulation (Sreenivas et al., 2012; Sharaev et al., 2016; Yang et al., 2020); more negative emotions and poor emotional regulation are two of the main characteristics of depression (Silk et al., 2003; Young et al., 2019). Moreover, recent a meta-analysis revealed that the dmPFC is associated with rumination, which is another main characteristic of depression (Roelofs et al., 2008). Although previous VBM researchers have found reduced GMV in the dmPFC of clinically depressed patients (Frodl et al., 2008), our results found increased GMV in the dmPFC of healthy subjects. We believe that one of the important reasons for the inconsistent results may be that healthy subjects were used in this study. Although individuals with high scores on the SDS were more depressed, they were still healthy subjects (Wang et al., 2015). Therefore, the correlation between the volume of the dmPFC and depressed emotion found in this study may indicate that individuals with high levels of depressed emotion and a large GMV in the dmPFC may have abnormalities in self-reference, emotional processing, emotional regulation and rumination.

The mediation analysis revealed that the relationship between neuroticism and depressed emotion was partially mediated by the GMV of the dmPFC at T1. As discussed above, high levels of neuroticism affect the GMV of the dmPFC, which are related to self-reference, emotion processing, emotion regulation, and rumination (Northoff et al., 2006; Etkin et al., 2011; Yang et al., 2020; Zhou et al., 2020). Depressed individuals have more negative self-reference, more negative emotion, more rumination, and poor emotion regulation (Wisco, 2009; Disner et al., 2011; Joormann and Quinn, 2014). The results of this mediation analysis may indicate that neuroticism influences depressed emotion *via* the GMV of the dmPFC. Additionally, the partial mediation may be due to the involvement of other important brain regions in neuroticism and depressed emotion that were not examined in this study. The results did not indicate that GMV in the dmPFC mediated the relationship between neuroticism and depressed emotion at T2. Although neuroticism scores (T1) were still significantly associated with depressed emotion scores 6 months later (T2), individuals may experience much uncertainty or stressful life events over the course of 6 months. Previous longitudinal study has demonstrated that life events, especially stressful life events, can affect brain structure within just 3 months (Papagni et al., 2011). For example, researchers found that the GMV of the prefrontal cortex decreased in those who experienced stressful sexual events (Ansell et al., 2012; Ringwald et al., 2022). Another study found that individuals who had experienced childhood maltreatment have significantly increased GMV in the dmPFC (Chaney et al., 2014). Life events that have occurred in the 6 months between T1 and T2 may have affected the brain structure of the dmPFC, thus inhibiting the mediating effect at T2 in present study.

There are some limitations in present study. First, the levels of depression and neuroticism were self-reported by participants, without any statistical experimental procedure or design. Second, the ROI was considered the base for mediation analysis, and the small effect size may limit the generalizability of the results. Third, there were no MRI data or behavioral data (e.g., life events data) at T2, thus limiting the conclusion we can draw from our findings.

## Conclusion

The dmPFC volumetric changes examined herein could partially explain the cognitive functioning effects and the heightened negative emotions among individuals with higher levels of neuroticism. Furthermore, the mediation analysis also indicated that the dmPFC could be critical to the relationship between neuroticism and depressed emotion.

## Data availability statement

The original contributions presented in the study are included in the article/Supplementary material, further inquiries can be directed to the corresponding authors.

## Ethics statement

The studies involving human participants were reviewed and approved by Brain Imaging Center Institutional Review Board of Southwest University (China). The patients/participants provided their written informed consent to participate in this study.

## Author contributions

JY contributed to designing experiments, data analysis, and writing this manuscript. XH, DT, and AH contributed to data collection and manuscript revision. All authors contributed to the article and approved the submitted version.

## Funding

The National Natural Science Foundation of China (grant number 31800947) and Nanhu Scholars Program for Young Scholars of Xinyang Normal University funded this study.

## Conflict of interest

The authors declare that the research was conducted in the absence of any commercial or financial relationships that could be construed as a potential conflict of interest.

## Publisher’s note

All claims expressed in this article are solely those of the authors and do not necessarily represent those of their affiliated organizations, or those of the publisher, the editors and the reviewers. Any product that may be evaluated in this article, or claim that may be made by its manufacturer, is not guaranteed or endorsed by the publisher.
